# Read Length and Repeat Resolution: Exploring Prokaryote Genomes Using Next-Generation Sequencing Technologies

**DOI:** 10.1371/journal.pone.0011518

**Published:** 2010-07-12

**Authors:** Matt J. Cahill, Claudio U. Köser, Nicholas E. Ross, John A. C. Archer

**Affiliations:** 1 Department of Genetics, University of Cambridge, Cambridge, United Kingdom; 2 Department of Chemical Engineering and Biotechnology, University of Cambridge, Cambridge, United Kingdom; 3 Division of Chemical and Biological Engineering, Computational Bioscience Research Center, King Abdullah University of Science and Technology, Thuwal, Saudi Arabia; Duke University Medical Center, United States of America

## Abstract

**Background:**

There are a growing number of next-generation sequencing technologies. At present, the most cost-effective options also produce the shortest reads. However, even for prokaryotes, there is uncertainty concerning the utility of these technologies for the *de novo* assembly of complete genomes. This reflects an expectation that short reads will be unable to resolve small, but presumably abundant, repeats.

**Methodology/Principal Findings:**

Using a simple model of repeat assembly, we develop and test a technique that, for any read length, can estimate the occurrence of unresolvable repeats in a genome, and thus predict the number of gaps that would need to be closed to produce a complete sequence. We apply this technique to 818 prokaryote genome sequences. This provides a quantitative assessment of the relative performance of various lengths. Notably, unpaired reads of only 150nt can reconstruct approximately 50% of the analysed genomes with fewer than 96 repeat-induced gaps. Nonetheless, there is considerable variation amongst prokaryotes. Some genomes can be assembled to near contiguity using very short reads while others require much longer reads.

**Conclusions:**

Given the diversity of prokaryote genomes, a sequencing strategy should be tailored to the organism under study. Our results will provide researchers with a practical resource to guide the selection of the appropriate read length.

## Introduction

Since the first published study using 454's next-generation sequencing technology [1], a number of competing technologies have become available or will soon be released. These include platforms from Applied Biosystems, Helicos, Illumina, and Pacific Biosciences [2].

Arguably, the high throughput and relatively low per-base cost of any of these next-generation technologies should allow individual researchers to generate sequence data of sufficient depth to accurately determine the sequence of a prokaryote genome. However, producing a complete and finished sequence remains a challenge. Regardless of the technology, shotgun reads are assembled to produce a collection of contigs, separated by gaps that must be closed manually.

One way in which the competing sequencing platforms differ is in the length of the reads they produce. There is scepticism surrounding the technologies that produce very short reads (<50nt), particularly in the context of the *de novo* assembly of a whole genome [2], [3], [4], [5], [6]. This reflects an expectation that short reads will be unable to resolve small, but presumably abundant, repeats [7].

Importantly, the technologies that produce the shortest reads also offer the highest throughput and lowest per-base cost [4]. Researchers are therefore compelled to weigh the improved assembly results to be expected from longer reads against the cost savings offered by the very short reads. Although read pairing can, to an extent, compensate for read length, this also comes with an increased cost per-base [8]. Thus, there is no guarantee that simply opting for the longest available read or mate pair will be the most cost-effective strategy.

To be clear, reads of 1000nt in length, or a mate pair separated by the same distance, will produce a more complete assembly than unpaired 75nt reads. But by how *much*? If the former options produced an assembly consisting of 20 contigs and the latter 30, it would be difficult to justify any additional costs for the longer reads or pairing. While the particular figures in this example are optimistic, the actual relationship between read length and the frequency of unresolvable repeats is not clear. Likewise, it is not known how this varies among prokaryotes. It is likely that for some species, very short reads would be sufficient, whereas others would require longer reads to produce an assembly of similar quality.

This work is intended to address this general question. It is hoped that by providing a concrete assessment of the value of read length, in terms of the ability to resolve repeats, researchers will be able to better judge the relative merits of the various sequencing options that are available.

Several previous studies have assessed the feasibility of sequencing using very short reads, focussing particularly on the challenge posed by repeat resolution [9], [10]. However, these involved only a small number of species and thus did not explore the diversity of prokaryote genomes. In contrast, Kingsford et al. (2010) incorporates a large selection of genomes, but focussed on providing a benchmark for evaluating assembler performance and the ability of short reads to reconstruct complete genes [8].

In common with Kingsford et al. (2010), this study examines the limit imposed by repeat resolution in a large number of genomes. However, our focus is the production of complete sequences. While a draft consisting of a collection of contigs is often sufficient for some applications, a complete sequence is, for a variety of reasons, more desirable [11], [12]. Further, it is a comprehensive record of the structure and content of a genome and will remain a useful resource for many years. The same cannot be said of an incomplete draft.

Using a simple model of repeat assembly, we develop and test a technique that, for any read length, can estimate the occurrence of unresolvable repeats in a sequenced genome and thus predict assembly results. We then apply this technique to 818 prokaryote genome sequences. This provides a quantitative assessment of the relative value of various read lengths, in terms of their ability to resolve repeats and produce readily finished assemblies. We go on to explore the relationship between read length and repeat resolution in greater detail in a subset of genomes. Taken together, this work provides interested researchers with a practical resource to guide future sequencing projects.

## Results

### The problem posed by repeats

Gaps in an assembly may arise as a result of repeated sequences or because a region of the genome is simply not represented in the read set (i.e. a sequence gap). An implicit assumption of this work is that the former will be largest, if not sole contributor, to the fragmentation of an assembly. We explored the validity of this assumption by analysing a set of error-free 36nt Illumina sequencing reads from the 4.6 Mb *Escherichia coli* K12 MG1655 genome (see Supplementary Methods (File S1)).

**Figure 1:**
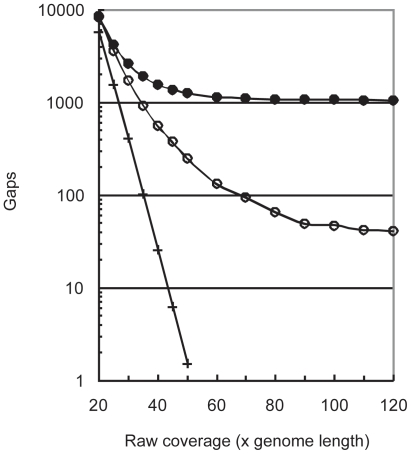
Assessing the cause of gaps in an assembly of 36nt reads. The predicted number of sequence gaps based on the Lander-Waterman model (+) is presented along with the actual number of sequence gaps in sets of 36nt Illumina reads (○). This was determined by aligning the reads in each set to the reference sequence. The total number of gaps present in Velvet assemblies of the various read sets is also included (•). The numerous additional gaps observed in the assemblies are due to unresolvable repeats (○ vs. •). Additional details can be found in the Supplementary Methods (File S1).

Traditionally, the model of Lander-Waterman [13], [14] is used to predict the number of sequence gaps in an assembly. The model predicts that for 36nt reads a raw coverage depth of approximately 50× would allow for the complete reconstruction of the *E. coli* genome (Figure 1). To determine the actual number of sequence gaps in the Illumina read set we compared the reads to the reference genome using BLAST [15].

At any given depth of coverage, the Illumina read set has a greater number of sequence gaps than predicted by Lander-Waterman. This is not surprising as a real-world sequencing technology cannot be expected to produce the random distribution of reads which is assumed by the Lander-Waterman model. However, when the same data sets are processed with Velvet, the resulting assemblies are far more fragmented than we would expect from sequence gaps alone [16]. At 120× coverage, the assembly consisted of 1,054 contigs. Only 37 of the gaps were due to missing data, whereas the remainder were associated with unresolvable repeats. For additional details of the sequence assembly and analysis refer to the Supplementary Methods (File S1).

That unresolvable repeats are *a* cause of gaps is not surprising. What this case does illustrate is that, in practice, essentially *all* gaps are associated with repeats. However, reaching this repeat-imposed limit requires excess coverage, although not beyond what is characteristic of a real data set. Thus, regarding unresolvable repeats as the principle cause of gaps in actual assemblies is justified.

In general, given the diversity of bacterial genomes, and the multitude of available technologies, it is not possible to identify a particular depth of coverage that would minimise sequence gaps. The most obvious approach would be to continue sequencing until no new data are produced. How this can be achieved will be technology-dependent.

### Predicting repeat-induced gaps

The obvious way to explore the relationship between read length and repeat resolution would be to carry out assemblies of simulated read sets of various lengths. However, this is a time-consuming process and we would therefore be limited to an arbitrarily selected set of read lengths and a small, but hopefully representative, collection of bacterial genomes.

Instead, we developed an algorithm that predicts assembly results based on the sequence of a genome and the number and average length of the sequencing reads. Briefly, exact repeat pairs are first identified using *repeat-match* from the MUMmer package [17], [18]. These results are then processed to produce a list of the repeat lengths, 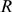
_,_ and their frequencies, 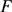. At this point, a simple model of repeat assembly is used to estimate the fraction of the repeats of each length which are not resolved. The sum of all of these unresolved repeats is the total number of repeat-induced gaps in the assembly.

**Figure 2:**
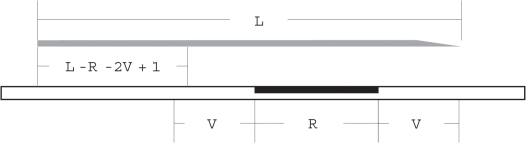
A model of repeat assembly. To unambiguously assemble a repeat (black rectangle), a read must encompass the entirety of the repeat and extend, in both directions, into unique sequence. If the repeat has a length of 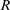 nt, and the adjacent unique sequence must be at least 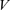 nt, then resolution of the repeat requires that a read starts in a 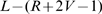 window next to the repeated sequence. The likelihood of this failing to occur in an assembly of a given number of reads of a particular length, can be estimated using an approach analogous to that used to compute sequence gaps [13], [14].

To guarantee the correct assembly of a repeated sequence, at least one read must encompass the entirety of the repeat, and extend in both directions into adjacent unique sequence. In Figure 2, the length of the reads and of the repeat are 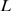 and 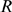, respectively. To assemble the repeat, the read must extend 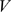 bp into the unique flanking regions. The extent to which the read must overlap the flanking sequence will depend on the particulars of the assembly. In theory, only a single nucleotide either side of the repeat would be sufficient. This is what is assumed in subsequent analyses.

There are two conditions under which a repeat will be unresolvable. Firstly, if the read length is less than the sum of the repeat length and required overlaps, then the repeat cannot possibly be resolved with the available reads. Assembly of a repeat therefore requires that 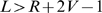. Secondly, even with sufficient read length, a gap will result if, by chance alone, none of the reads in the shotgun dataset actually span the repeat. In Figure 2, a read must begin in a window of 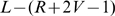 bp adjacent to the repeated region to allow assembly. The likelihood of this *not* happening can be estimated using a model analogous to that of Lander-Waterman [13], [14].

For a genome of length 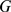, the probability that a given read does not start in the window 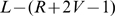 is 
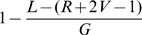
. For 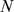 shotgun reads, the probability that no read starts in the window is then
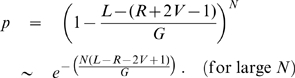
(1)Using (1), and the repeat length frequencies discussed above, we can predict the total number of gaps in any assembly. Specifically, for a given repeat length 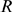 occurring 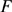 times in the genome, the number of unresolved repeats is 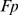 when 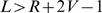 or simply 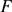 when 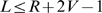. The total number of repeat-induced gaps expected in the assembly is the sum of the unresolved repeats of each length.

A key difference between our model of assembly and how assemblers actually operate lies in how unresolvable repeats contribute to the total number of contigs. In our model, unresolvable repeats only cause gaps. In reality, in addition to causing a gap, a contig may be produced which corresponds to the repeat itself (Figure S1). The algorithm therefore predicts the number of *unique* contigs and gaps between them, rather than the total. The former is a more useful figure as finishing requires only that the unique contigs be joined. Any contigs which correspond to repeats would be addressed as a necessary corollary of this.

### Assessing the accuracy of the algorithm

To ensure that the algorithm was predictive for a variety of organisms and a broad range of read lengths, we compared its predictions to actual assemblies of *Mycoplasma genitalium* (580 kb), *E. coli* K12 MG1655 (4.6 Mb), and *Streptomyces coelicolor* (8.7 Mb), at five different read lengths: 36, 75, 125, 250, and 500nt. These read sets were simulated.

**Figure 3:**
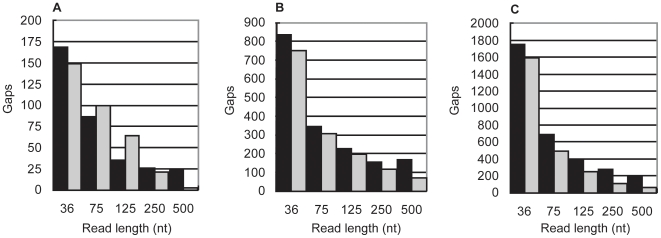
Assessing the accuracy of the algorithm. The number of repeat-induced gaps predicted by the algorithm (grey bars) compared to the number of gaps observed (black bars) in actual assemblies of 36, 75, 125, 250, and 500nt simulated reads from **A**) *M. genitalium*, **B**) *E. coli* and **C**) *S. coelicolor*. The observed gaps are those between unique, non-redundant contigs larger than the read length. The coverage depth of each read set was the threshold at which random gaps are no longer predicted by the Lander-Waterman model. This occurs at effective coverage depths of 9–17×.

In Figure 3, the number of gaps between unique, error-free, contigs in each assembly is presented along with the predicted number of gaps based on the algorithm. If included, the error-containing contigs would have increased the total number of contigs by only 3.4% in the most extreme case.

The algorithm accurately predicts the vast differences between the genomes in terms of the number of gaps observed at a given read length. In addition, for each genome, the algorithm is broadly predictive of the overall relationship between read length and gap number. However, its performance is not consistent for all variables. The predictions for *M. genitalium* are generally less accurate than those for the other genomes. This likely reflects the small size of the *M. genitalium* genome. A relatively modest divergence, in absolute terms, appears significant when the total number of gaps is small.

### Read length requirements amongst prokaryotes

One way to look at the relative value of various read lengths is to evaluate their performance on a large number of prokaryote genomes. We applied our algorithm to 818 complete prokaryote chromosomes (downloaded from GenBank, June 2009). For each sequence we determined the number of repeat-induced gaps at a number of read length benchmarks. These were: 36, 75, 125, 250, 500, and 1,000nt. Using a reciprocal approach, the algorithm was used to calculate the read length required to produce assemblies with 48, 96, 192, 384, and 762 gaps (gap benchmarks). These particular values were selected because they are convenient multiples of the microtitre plate sizes that might be used during the finishing phase of a project. A full list of the benchmark data for all 818 genomes is provided in Table S1.

**Figure 4:**
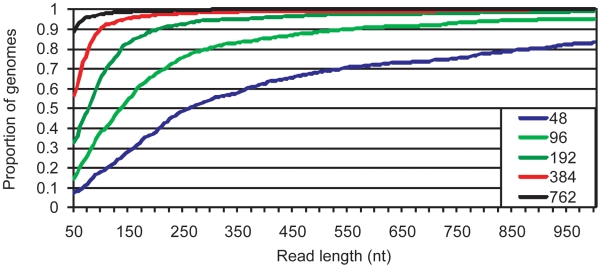
Assessing the performance of a range of read lengths. The fraction of the 818 genomes that meet gap benchmarks as a function of read length was calculated. The benchmarks were 762, 384, 192, 96, and 48 repeat-induced gaps. For example, assuming reads of 150nt, 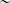
50% of the genomes can be assembled with fewer than 96 gaps.

In Figure 4, the proportion of the 818 genomes that would meet the various gap benchmarks as read length increases is depicted. For 75% of the genomes analysed, a read length of only 245nt is enough to produce assemblies with fewer than 96 repeat-induced gaps. Reads of 125 and 70nt are sufficient for the same percentage of genomes to meet the 192 and 384 gap benchmarks, respectively.

### Relationship between read length and repeat resolution

The 818 genomes included in this analysis encompass the overwhelming majority that have been sequenced to date. However, they are surely not a representative sample of this domain, being biased towards culturable organisms and pathogens in particular. Nonetheless, the preceding analysis does provide an initial estimate of the relative value of various read lengths when sequencing prokaryotes generally. It also illustrates that there is considerable variation in read length requirements.

**Figure 5:**
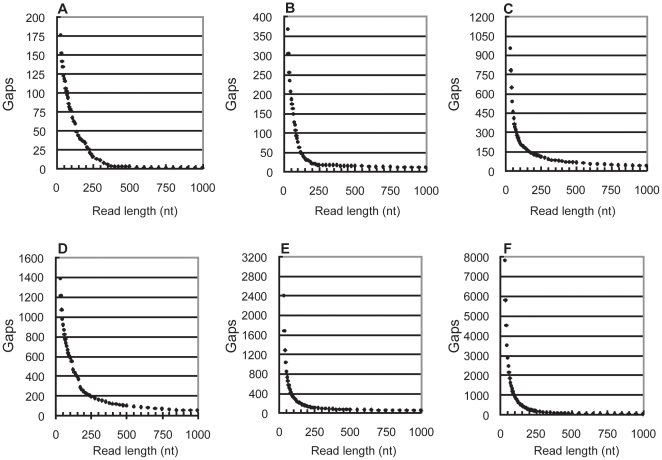
Read length and repeat resolution in 6 genomes. The algorithm was used to predict the occurrence of repeat-induced gaps in assemblies of six bacterial genomes from a range of read lengths. A raw coverage of 100× was used for all genome/read length pairings. Assembly results were predicted for read lengths at increments between 30–1,000nt. Between 30 and 100nt the increment was 5nt; 100–250nt, 10nt; 250–500nt, 25nt; and 500–1,000nt, 50nt. **A**) *M. genitalium* (580 kb), **B**) *H. Influenza* (1.8 Mb), **C**) *E. coli* (4.6 Mb), **D**) *N. meningitidis* (2.3 Mb), **E**) *S. coelicolor* (8.7 Mb) and **F**) *S. cellulosum* (13.0 Mb).

To determine how the relationship between read length and the frequency of unresolvable repeats varies, a detailed analysis of a subset of genomes was carried out. *M. genitalium* (NC_000908.2), *E. coli* (NC_000913.2), *Haemophilus influenzae* (NC_000907.1) and *S. coelicolor* (NC_003888.3) were analysed to provide a range of genome lengths. The extremely large genome of *Sorangium cellulosum* (NC_010162.1), and the repeat-rich genome of *Neisseria meningitides* (NC_003112.2) were also included [19]. The predicted assembly results, assuming 100× raw coverage at every length, are presented in Figure 5.

First, very short reads (<50nt) produced highly fragmented assemblies. This agrees with intuitive expectations, has been observed previously, and is therefore unsurprising [16], [20], [21], [22], [23], [24]. However, what is notable is that, although extending read length undoubtedly improves the assembly, the magnitude of the improvement shrinks consistently as reads grow. Using *E. coli* as an example, increasing the read length from 50 to 100nt closes 244 gaps; from 250 to 300nt, only 18. Once a relatively modest read length is reached, 250nt for example, the overwhelming majority of repeated sequences in all of the genomes have been resolved.

It was thought that the presence of multi-copy repeat families would cause a relative abundance of repeats of a particular length [25]. Extending the reads beyond this threshold length would cause a sudden improvement in the assembly and introduce a step-wise character to the curves in Figure 5. This does not appear to be the case for the bacterial genomes examined here. The most probable explanation is that the algorithm only identifies exact repeats. Members of a repeat family which have diverged would be detected as a series of small exact repeats rather than a single, large, degenerate repeat. As such, there would be no accumulation of repeats at a specific length that would be required to produce a “step” in the curves. Given the demonstrated accuracy of the algorithm, the coverage depths achievable with next-generation sequencers, and the underlying characteristics of short read assemblers, the assumption that only exact repeats are problematic is probably justified. Thus, it is unlikely that significant length thresholds would be observed in true assemblies.

### Guidance for read length selection

The graphs in Figure 5 illustrate that the overall relationship between read length and contiguity of an assembly is broadly consistent among bacteria. However, the absolute number of gaps at a particular read length varies by as much as an order of magnitude. For example, 75nt can reconstruct the *M. genitalium* genome with only 97 gaps whereas the corresponding figure for *S. cellulosum* genome is almost 1,500. Therefore, the most cost-effective sequencing strategy depends on the particular organism. This is problematic as little can be known about the repeat content of a genome prior to sequencing. Thus, tailoring the strategy has to be done based on some readily available characteristic of the organism.

Intuitively, genome length seems to be such a characteristic. However, there is a poor correlation between genome length and the frequency of unresolvable repeats given a particular read length (Figure S2 [8]).

Assuming that closely related genomes would be comparably difficult to sequence and assemble, it would be logical to use available sequences to help select a read length for the organism of interest. As an example, researchers might sequence large collections of clinical isolates or additional species from industrially important genera.

**Figure 6:**
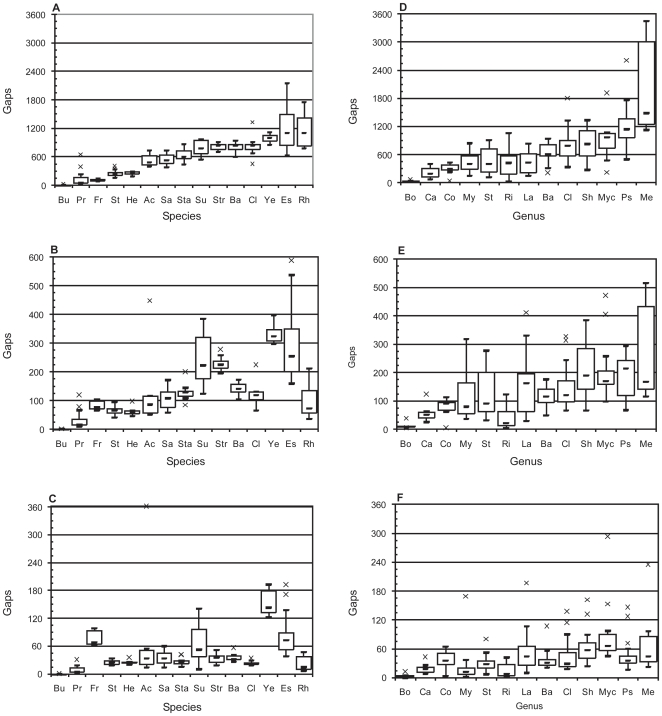
Variation in assembly results within taxa. The median number of repeat-induced gaps for all members of a group is represented by (−). The lower and upper bounds of the hollow rectangle correspond to the first and third quartile, and the range is indicated by the whiskers. Any outliers are plotted as (×). In **A**)–**C**), the species are are ***Bu*** *chnera aphidicola*, ***Pr*** *ochlorococcus marinus*, ***Fr*** *ancisella tularensis*, ***St*** *reptococcus pyogenes*, ***He*** *licobacter pylori*, ***Ac*** *inetobacter baumannii*, ***Sa*** *lmonella enterica*, ***Sta*** *phylococcus aureus*, ***Su*** *lfolobus islandicus*, ***Str*** *eptococcus pneumoniae*, ***Ba*** *cillus cereus*, ***Cl*** *ostridium botulinum*, ***Ye*** *rsinia pestis*, ***Es*** *cherichia coli*, ***Rh*** *odopseudomonas palustris*. In **D**)–**F**), the genera are ***Bo*** *rrelia*, ***Ca*** *mpylobacter*, ***Co*** *rynebacterium*, ***My*** *coplasma*, ***St*** *reptococcus*, ***Ri*** *ckettsia*, ***La*** *ctobacillus*, ***Ba*** *cillus*, ***Cl*** *ostridium*, ***Sh*** *ewanella*, ***Myc*** *obacterium*, ***Ps*** *eudomonas*, ***Me*** *thylobacterium*. *For Methylobacterium*, outliers at 36nt (6,307) and 125nt (1,219) have been omitted. Gap predictions are for reads of **A**)/**D**) 36nt, **B**)/**E**) 125nt, and **C**)/**F**) 500nt.

To provide an estimate of the reliability of this approach, the benchmark data were analysed to determine the variation in assembly results within recognised species and genera. The 818 genomes included in the analysis were searched to identify those species for which 6 or more isolates have been sequenced. The variation in assembly results among isolates of the same species is depicted in Figure 6 A–C.

Not surprisingly, the species differ considerably in the median number of repeat-induced gaps predicted at any read length. However, for most species, the range is small enough that a single sequencing strategy would be appropriate for all members. For example, very short reads (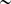
36nt) are probably sufficient for isolates of species *Buchnera aphidicola* and *Francisella tularensis*, whereas longer reads (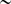
125nt) are required for *Prochlorococcus marinus*, *Streptococcus pyogenes*, *Helicobacter pylori*, *Salmonella enterica*, *Staphylococcus aureus*, and *Bacillus cereus*.

An identical analysis was conducted on those genera for which 6 or more distinct species have been sequenced (Figure 6 D–F). Not surprisingly, the typical range of assembly results was larger for the genera than for the species. Nonetheless, devising a sequencing strategy on the basis of a previously sequenced member of the same genus is probably justified.

In Figure 6, there are some groups for which the assembly results are noticeably more variable than others; a familiar example being *E.coli* (Figure 6 A–C). This is not surprising as in this analysis, organisms were grouped together solely on the basis of a common species or genus name. Avoiding the ongoing debate as to the significance, if any, of a bacterial species, it is enough to say that the actual degree to which the various genomes in each group have diverged is not consistent [26]. Thus, the results depicted in Figure 6 are, at best, an estimate of variation within recognised, though necessarily arbitrary, groups of organisms.

In practice, it would be sensible to rely on the closest available relative in Table S1 when devising a sequencing strategy rather than the median or average for a group. Returning to the *E.coli* example, it is the presence of several large and repeat rich O157:H7 genomes that skew the results for this species [27]. If the goal was to sequence a variety of O157:H7 clinical isolates, the sequencing strategy should reflect the benchmark data from the O157:H7 genomes rather than the lab strains.

## Discussion

The motivation for this work was the belief that, with many sequencing options available, a precise understanding of the value of read length, in terms of repeat resolution, is necessary to select the best technology for a particular application.

Our results confirm that, for at least one of the technologies, repeats *are* effectively the sole cause of assembly gaps, but only when excessive coverage is used. It is our view that targeting excess coverage is justified if a complete sequence is the objective. Primarily, this reflects changes in the relative costs of the shotgun and finishing phase of a genome project. In the *E. coli* example using 36nt reads, increasing the raw coverage from 40× to 100× would increase the sequencing costs by a factor of 2.5 without changing the duration of this phase. The result would be 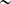
500 fewer sequence gaps requiring closure. In our experience, closing these gaps “manually” would likely cost more than the additional sequencing reads and would substantially increase the duration of the entire project.

In addition, the principal obstacle encountered during gap closure is establishing the order of contigs. Once this is determined, closing gaps using sequenced PCR products is a trivial matter. In this context, sequence gaps and those that are caused by repeats fundamentally differ. Without supplementary data, ordering contigs that are separated by sequence gaps presents an enormous combinatorial problem. In contrast, repeat-induced gaps exist because there are at least two equally acceptable ways to assemble a repeated sequence. In this case, the order of contigs is constrained by their relationship to repeats. This distinction is a further incentive to minimise the occurrence of sequence gaps.

Until recently, the relative value of various read lengths, in terms of their ability to resolve repeats, was not clear. Intuitively, longer reads are preferable and individual researchers may have arrived at their own conclusions about what lengths were suitable for prokaryote sequencing. Our analysis of the 818 genomes constitutes a systematic assessment of the performance of a range of read lengths. In practice, this analysis demonstrates that relatively modest lengths can produce well-connected assemblies for the majority of prokaryotes.

A more detailed examination of the relationship between read length and repeat resolution in a small set of genomes reveals that extending reads produces consistently diminishing returns and this appears to be consistent among bacteria.

Nonetheless, amongst prokaryotes, there exists considerable variation in the absolute number of gaps at a given read length, and genome length is a poor predictor of this. Thus, selecting a sequencing technology for a particular organism is probably best done with reference to the benchmark values of a sequenced relative (Table S1). Broadly, the relatively small differences in the frequency of repeat-induced gaps among isolates of the same species validates this approach (Figure 6).

Although not explicitly addressed in this work, paired reads can be conservatively regarded as *pseudo*-reads with a length equal to that of the template molecule. Consequently, our results can be extended to sequencing with read pairing. It is tempting to assume that, with few exceptions, the largest possible mate pair would be appropriate sequencing strategy for all prokaryotes. However, increasing the distance between paired reads is not without drawbacks [16]. Our results can be used to select the minimum insert length that produces a readily finished assembly. This might also help avoid the need for multiple libraries [28]. Furthermore, our results show for which species unpaired sequencing is adequate, thus avoiding the additional costs and technical challenges associated with paired sequencing altogether [8].

The utility of our results is primarily to provide the scientific community with a practical resource that should allow for a more rational approach to prokaryote genome sequencing. A researcher can examine the available sequencing options in light of the benchmark data, select a technology, and have some confidence as to the characteristics of the resulting assembly. Hopefully this will lead to the production of more *complete* sequences using next-generation platforms, rather than unfinished collections of contigs.

At present, the per-base cost of sequencing tends to increase with increasing read length. Given the rate at which sequencing technology is developing it would invite embarrassment to predict how long this condition will persist. There are already technologies under development which promise multi-kb read lengths [2], [29]. However, until something better comes along, researchers will be compelled to balance cost against contiguity. The results presented here should remove some of the uncertainty from this decision.

## Methods

### The algorithm - description

The algorithm used to predict assembly results was as follows: First, r*epeat-match* from the MUMmer package [17], [18] was used to identify exact repeat pairs, regardless of their orientation. The minimum length threshold that was used was 22bp, unless otherwise stated. The pairwise results were processed to produce a list of all of the identified repeats and their location in the genome. These data were then sorted by location and nested, bordering, or partially overlapping repeats were merged. Finally, the repeat content of the genome was reduced to a table of merged repeat lengths and the number of times they were observed.

Using these data, the fraction of repeats of each length left unresolved, given a particular read length and coverage depth is then determined. The formula used to compute the unresolved fraction is 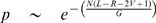. The derivation of this formula is discussed in the results section. Throughout this work, 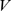 is set to 1 bp. For convenience, we refer to the raw coverage provided by various read sets, e.g. 100×. The total number of reads, 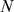, is computed by multiplying this value by the genome length, 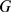, and dividing the product by the read length, 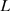.

### The algorithm - validation

Generating the simulated read sets was a two step process. First, a list was created that defined the length, strand, and start location in the genome of a large number of reads. The latter two parameters were randomly generated using the Perl module Math::Random. For the 36, 75, and 125nt read sets, a uniform read length was assumed. When generating the 250 and 500nt reads, the lengths was randomly sampled from a normal distribution with the appropriate mean, 250 or 500nt, and standard deviations taken from real 454 data (SRR01355 and SRR014812).

The simulated reads were generated by extracting sequences from the genome based on these parameters. Reads were reverse-complemented if they were from the opposite strand. To simplify subsequent analyses, any reads that would extend beyond the edges of the genome were discarded. For a given read length/genome pairing, a very large set of reads was first constructed and then reads were sampled from this set to produce defined coverage subsets.

In terms of raw coverage, the sizes of the 36, 75, 125, 250, and 500nt read sets were as follows: For *M. genitalium*, 105×, 22×, 16×, 12×, and 10×, respectively. For *E. coli*, 120×, 26×, 18×, 15×, 13×. For *S. coelicolor*, 125×, 26×, 20×, 16×, 14×.

Unless otherwise stated, Velvet version 0.7.31 was run with default settings. When processing 36nt reads, the “-short” flag was used. All other assemblies were run with “-long”. In all cases, the hash length was set to 31, *max_divergence* set to 0, and *long_mult_cutoff* set to 10. An extensive range of parameter combinations was tested and these were found to produce the best assembly results (data not shown).

After assembly, contigs were processed as follows: First, BLASTN was used to align a contig set to both itself and to the reference genome (-m 8 -e 1E-10 -F f). Any error-containing contigs - those without perfect alignments to the reference - were removed from consideration at this stage. In addition, any contigs that were nested entirely within a larger contig were assumed to be assembler errors and were discarded. Finally, those contigs that were shorter than the reads used in the assembly were removed and a series of summary statistics were computed for those that remained. This included the number of unique contigs, the total number of contigs, and the portion of the genome that was covered by the latter. The number of gaps between unique contigs in each of the assemblies was compared to the predictions of the algorithm.

### Sequencing benchmarks

The genome sequences were downloaded from ftp://ftp.ncbi.nih.gov/genomes/Bacteria/ on June 14, 2009. This dataset was searched to identify those sequences which contained “complete genome” in their description. This identified 876 sequences among the 894 taxa included in the directory. Genomes consisting of more than one chromosome were discarded as multiple sequences cannot be processed by *repeat-match*. As well, several sequences from extrachromosomal elements were identified and removed. This left 818 complete prokaryote genome sequences. A complete list of the genomes analysed is in included in Table S1.

For the length benchmarks, the algorithm was used to predict assembly results for 36, 75, 125, 250, 500, and 1,000nt reads. The gap benchmarks were determined by first predicting assembly results for read lengths at 5nt increments between 30–1,000nt. These data were then processed to identify the lengths at which the number of repeat-induced gaps fell below each of the benchmarks (48, 96, 192, 384, and 762 gaps). In all cases, 100× raw coverage was assumed.

The species and genera that were used to assess variation in assembly results among related genomes were selected based on the following criteria. A species was included in the analysis if there were at least 6 genome sequences from organisms with that name. A genus was included if there were at least 6 genomes from the group and that these were derived from *different* species. Data points were considered outliers if they did not fall within 1.5 times the inter quartile range.

## Supporting Information

File S1 Supplementary methods.
(0.03 MB PDF)

Figure S1 The algorithm predicts the occurrence of repeat-induced gaps, rather than the total number of contigs produced in an assembly. In this example, a genome containing 2 repeat pairs (A and B) separated by stretches of unique sequence (U1–U5) is depicted. If the read length under consideration could not possibly resolve the repeats, the model would predict 4 gaps, and thus 5 unique contigs. In a true assembly, the repeats themselves may emerge as contigs, bringing the total number of contigs to 7.
(0.71 MB TIF)

Figure S2 The predicted number of repeat-induced gaps as a function of genome length. The results for 818 prokaryote genomes are depicted assuming reads of A) 36nt, B) 125nt, and C) 500nt. A raw coverage of 100× is used for all genome/read length pairings.
(1.06 MB TIF)

Table S1 Read length and gap benchmarks for 818 bacterial genomes. To produce the benchmark data in the left-most portion of the table, the algorithm was used to predict the number of repeat-induced gaps at six specified read lengths. The benchmarks on the right were produced by first specifying the maximum number of gaps an assembly could contain, for example, 96, then the algorithm was used to search for the shortest read that was predicted to produce an assembly that met this criterion.
(0.29 MB XLS)
