# Supplementary material for: Read Length and Repeat Resolution: Exploring Prokaryote Genomes Using Next-Generation Sequencing Technologies
Source: PLoS One. 2010 Jul 12;5(7):e11518. doi: 10.1371/journal.pone.0011518 (PMC2902515; doi:10.1371/journal.pone.0011518)
Supplement: File S1 — Supplementary methods. (0.03 MB PDF) [file pone.0011518.s001.pdf]

Read length and repeat resolution: Exploring prokaryote genomes using next-generation sequencing technologies.

Matt J. Cahill, Claudio U. Köser, Nicholas E. Ross, and John A. C. Archer

Supplementary Methods

Assembly of Illumina reads

A collection of sequencing reads from the *E. coli* K12 MG1655 genome were downloaded from the Short Read Archive at the NCBI (SRR001665). The 36 nt reads were generated as part of a paired-end sequencing run using an Illumina Genome Analyser.

BLASTN was used to identify any reads which did not align perfectly to the latest release of the genome sequence. Default parameters were used with the following exceptions: -e 1E-10, -F f. Any reads that did not align perfectly with the reference were discarded to simplify subsequent analyses. Of the 10,408,224 read pairs in the original data set, perfect alignments were found for 9,054,243 (86 %) forward reads and 8,621,037 (83 %) reverse reads. It is notable that the vast majority of the reads in this data set are completely error-free. In total, the error-free read set consisted of 7,749,398 read pairs corresponding to approximately 120 x raw coverage of the *E. coli* genome. Read pairs were sampled from this set to produce a range of defined coverage subsets (20-120 x).

Sequence gaps were identified by aligning reads to the reference genome using BLASTN (-e 1E-10, -F f). The coordinates of all perfect alignments for each read were tabulated then mock assembled by "joining" alignments which overlapped by at least 25 nt. This particular requirement reflects the hash length used in the true assemblies. Using this approach, sequence gaps appear as interruptions in the growing chain of overlapping alignments. As all perfect hits for a given read were identified, those reads that mapped to repeated regions would produce several perfect alignments.

The read sets were assembled using Velvet version 0.7.31 with default parameters, unless otherwise stated (Zerbino and Birney, 2008). The "-short" flag was used with a hash length of 25 and the exp_cov parameter was set to the effective coverage of the read set. To determine the cause of assembly gaps, the contigs were aligned to the genome sequence using BLASTN (-f F -e 1E-5) and the results were filtered to identify those hits that represented perfect alignments of an entire contig. The alignment coordinates of these contigs were then mapped to the locations of known repeats. A gap was determined to be repeat-associated if two neighbouring contigs ended within a repeated sequence. The technique used to identify repeats is described in the methods section. The minimum repeat length was set to 20 bp.
