# Supplementary figures and images for: Read Length and Repeat Resolution: Exploring Prokaryote Genomes Using Next-Generation Sequencing Technologies
Source: PLoS One. 2010 Jul 12;5(7):e11518. doi: 10.1371/journal.pone.0011518 (PMC2902515; doi:10.1371/journal.pone.0011518)

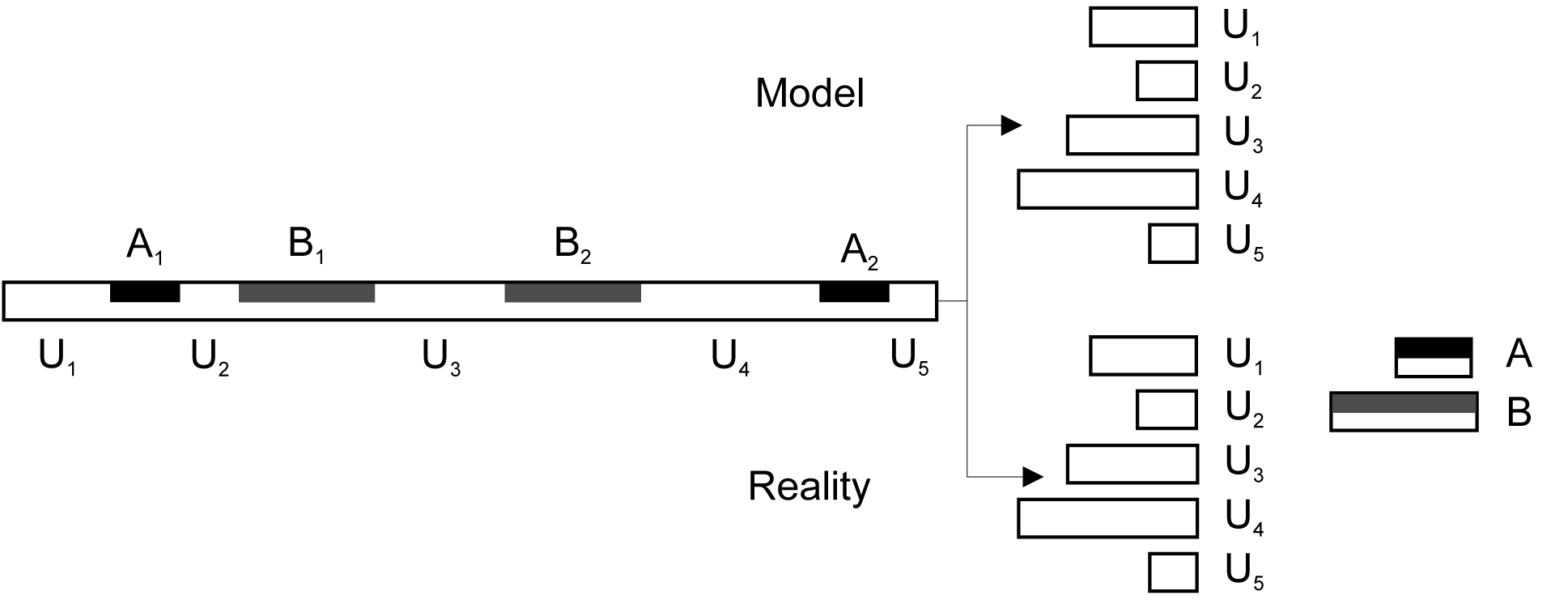

Supplement: Figure S1 — The algorithm predicts the occurrence of repeat-induced gaps, rather than the total number of contigs produced in an assembly. In this example, a genome containing 2 repeat pairs (A and B) separated by stretches of unique sequence (U1–U5) is depicted. If the read length under consideration could not possibly resolve the repeats, the model would predict 4 gaps, and thus 5 unique contigs. In a true assembly, the repeats themselves may emerge as contigs, bringing the total number of contigs to 7. (0.71 MB TIF) [file pone.0011518.s002.tif]

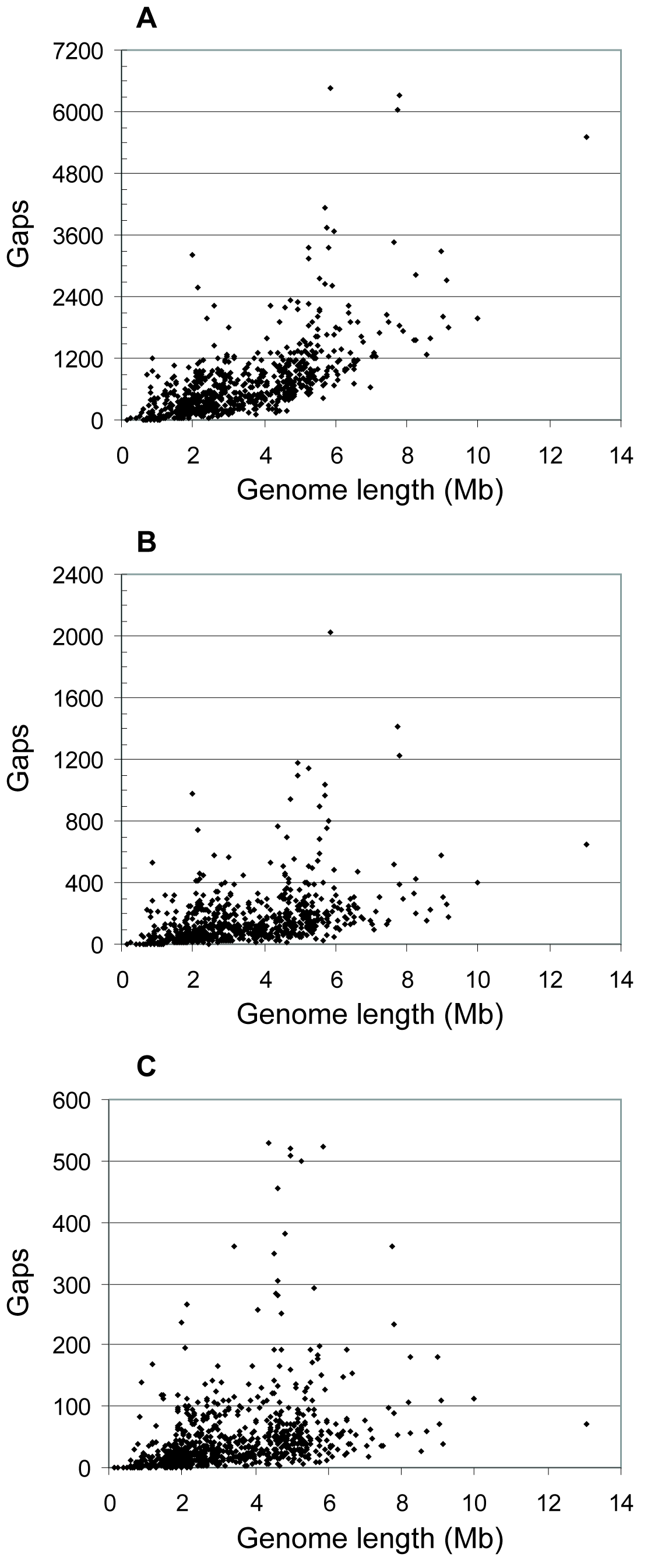

Supplement: Figure S2 — The predicted number of repeat-induced gaps as a function of genome length. The results for 818 prokaryote genomes are depicted assuming reads of A) 36nt, B) 125nt, and C) 500nt. A raw coverage of 100× is used for all genome/read length pairings. (1.06 MB TIF) [file pone.0011518.s004.tif]
